# Pregnant Women Hospitalized with Chikungunya Virus Infection, Colombia, 2015

**DOI:** 10.3201/eid2311.170480

**Published:** 2017-11

**Authors:** Maria Escobar, Albaro J. Nieto, Sara Loaiza-Osorio, Juan S. Barona, Fernando Rosso

**Affiliations:** Fundación Clínica Valle del Lili, Cali, Colombia (M. Escobar, A.J. Nieto, S. Loaiza-Osorio, F. Rosso);; Icesi University, Cali (M. Escobar, A.J. Nieto, J.S. Barona, F. Rosso)

**Keywords:** chikungunya virus, CHIKV, pregnancy, pregnant women, sepsis, critical care, newborn, arthritis, Colombia, epidemiology, South America, viruses, vector-borne infection, severe maternal morbidity, relapse, severe acute maternal morbidity, vertical transmission, chronic phase

## Abstract

In 2015 in Colombia, 60 pregnant women were hospitalized with chikungunya virus infections confirmed by reverse transcription PCR. Nine of these women required admission to the intensive care unit because of sepsis with hypoperfusion and organ dysfunction; these women met the criteria for severe acute maternal morbidity. No deaths occurred. Fifteen women delivered during acute infection; some received tocolytics to delay delivery until after the febrile episode and prevent possible vertical transmission. As recommended by a pediatric neonatologist, 12 neonates were hospitalized to rule out vertical transmission; no clinical findings suggestive of neonatal chikungunya virus infection were observed. With 36 women (60%), follow-up was performed 1 year after acute viremia; 13 patients had arthralgia in >2 joints (a relapse of infection). Despite disease severity, pregnant women with chikungunya should be treated in high-complexity obstetric units to rule out adverse outcomes. These women should also be followed up to treat potential relapses.

## Abstract 2

Personnel in charge of obstetric populations should watch for cases of chikungunya virus‒induced sepsis with hypoperfusion and organ dysfunction.

Chikungunya virus (CHIKV) is an alphavirus of the family *Togaviridae* that was isolated for the first time in Tanganyika (now Tanzania) in 1952 (*1*). CHIKV is transmitted to humans by several species of mosquito; *Aedes aegypti* and *Ae. albopictus* are the main vectors. Since 2004, the geographic distribution of the virus has expanded, which has led to major epidemics in Asia and Africa (*2*).CHIKV appeared as an emerging infection in the Americas in late 2013, with ≈1.1 million reported cases (*3*). In Colombia, by the 51st week of 2016, a total of 19,525 cases of CHIKV had been reported; 19,091 (97.8%) had symptoms suggestive of chikungunya, but only 206 (1.1%) had been confirmed positive for the virus by laboratory tests (*4*). Acute infection with CHIKV is characterized by high fever, asthenia, headache, emesis, rash, myalgia, and arthralgia (*1*,*5*). Infected persons usually recover spontaneously within several days or a week (*6*), but arthralgia can persist for months or even years (*7*).

Knowledge about CHIKV infection has been derived mostly from a large outbreak that occurred in Réunion Island in 2005. Therefore, data on the effects of this infection on maternal outcomes are limited, with no clear evidence that pregnant women infected with CHIKV have more obstetric complications. In a prospective study by Fritel et al., pregnant women with CHIKV infection were more likely to be hospitalized than nonpregnant women (*8*). Robillard et al. identified the first case of CHIKV vertical transmission (*9*), changing the perspective on perinatal infection. When maternal infection occurs at the end of pregnancy, serious, even life-threatening fetal and neonatal complications, such as meningoencephalitis and disseminated intravascular coagulation, can occur (*8*,*10*–*14*).

Information on the clinical presentation of chikungunya in pregnant women in Latin America is limited (*15*–*20*). The case descriptions in this report provide valuable information for the future management of suspected CHIKV infections during pregnancy, including information on the timing of delivery and appropriate level of care, which can help improve neonatal outcomes. The objective of this study was to describe the clinical features of the acute and chronic phases of CHIKV infection in pregnant women who were hospitalized in Fundación Valle del Lili, a fourth-level hospital. This hospital takes care of 1,400 births per year and serves as a reference facility for obstetrics cases of high clinical complexity for the southwest region of Colombia.

## Materials and Methods

### Type of Study

This investigation was a descriptive observational study of a series of cases. Pregnant women of any age who were hospitalized in the Unit of Obstetric High Complexity (UOHC) with CHIKV infection confirmed by reverse transcription PCR (RT-PCR) during January 1–December 31, 2015, were eligible for inclusion. All pregnant patients with RT-PCR‒confirmed infections were admitted to the UOHC, regardless of their clinical condition, so they could be better observed to rule out or manage possible adverse outcomes. We conducted a retrospective review of patient medical records to collect information on demographics, medical history, clinical findings, and laboratory results, as well as to review information recorded by the epidemiologic surveillance committee. This project was approved by the Biomedical Research Ethics Committee of the Fundación Clínica Valle de Lili according to ethics act number 25 of December 7, 2015.

### Laboratory Testing

To confirm the diagnosis of CHIKV infection, we performed RT-PCR in real time using the commercial LightMix kit Chikungunya-virus Light Cycler (TIB MOLBIOL, Adelphia, NJ, USA). We performed all CHIKV RT-PCR tests in the clinical laboratory of the Fundación Valle del Lili. Specific CHIKV genotypes were not identified in this study. However, Laiton-Donato et al. identified the genotypes responsible for the CHIKV epidemic in Colombia during 2014–2015 and found only the Asian genotype (*21*). We ruled out dengue virus infection by measuring blood for specific antibodies and nonstructural protein 1 antigen with the commercial SD BIOLINE Dengue Duo kit (Standard Diagnostic, Inc., Yongin, South Korea).

### Case Definitions

We stratified CHIKV infection into 2 phases: acute (first 10 days of disease) and chronic (after day 10 of disease) (*22*). We used the definition from the International Sepsis Definitions Conference in 2001 to standardize all cases of sepsis, severe sepsis, and septic shock (*23*). To assess severe acute maternal morbidity (SAMM) in pregnant women requiring intensive care, we decided to use the World Health Organization’s established criteria (*24*,*25*). To assess the chronic phase of disease, we contacted all patients by telephone 1 year after acute viremia. We used a survey used in previous studies (*16*,*26*) that included questions regarding the criteria for classification of rheumatoid arthritis of the American College of Rheumatology and European League of Rheumatism 2010 (*27*). The survey included questions on joint pain duration, morning stiffness, joint swelling, muscle pain, and joint redness. Qualified study authors (S.L.O. and J.S.B.) conducted the survey by telephone call (Technical Appendix Table).

### Data Collection

We reviewed electronic medical records to obtain information about pregnant women with CHIKV infection and newborns whose births were cared for at the Fundación Valle del Lili. We retrieved the following data: demographics, symptoms and duration of symptoms before hospitalization, findings from physical examination, findings from laboratory tests, and progress during hospitalization. We entered all information collected into an electronic database.

### Data Analysis

We performed a univariate analysis in which the distribution of numerical variables was evaluated by the Shapiro-Wilk test. We summarized data by using averages and SDs or medians and interquartile ranges (IQRs), as appropriate. We expressed qualitative variables as proportions and performed statistical analysis with the Stata program (StataCorp LLC, College Station, TX, USA).

## Results

We identified 60 patients with confirmed CHIKV diagnoses; all of these patients were hospitalized January–September 2015. No patients hospitalized during October–December 2015 were confirmed to have CHIKV infection. All 60 patients with confirmed CHIKV diagnoses were hospitalized at the OHCU to rule out sepsis and other viral infections, such as dengue. Mean patient age was 26.4 ± 5.6 years. Five (8.3%) women were in their first trimester (<12 weeks), 17 (28.3%) were in their second trimester (12–28 weeks), and 38 (63.3%) were in their third trimester (>28 weeks). Fifteen (25%) women pregnant in their third trimester completed pregnancy during the hospitalization, and 9 (15%) women required treatment in the intensive care unit (ICU).

### Clinical Findings

The most frequent clinical findings at the time of consultation at the emergency department were arthralgia (55/60, 91.6%), followed by fever (21/60, 35%) (Table 1). Other reported clinical manifestations were headache, rash, and myalgia. The interval between the onset of CHIKV symptoms and admission to the OHCU was 1.4 ± 0.5 days for patients in their first trimester, 1.4 ± 0.9 days for patients in their second, and 2.4 ± 1.9 days for patients in their third. We observed hemorrhagic manifestations among women in their third trimester (Table 1). Laboratory results showed leukopenia (<4,000 cells/µL) in 35 (58.3%) women and thrombocytopenia (<150,000/µL) and elevated transaminase levels (>32 µL) in 10 (16.7%) women. Thirty-one (51.7%) women had electrolyte abnormalities: 24 (77.4%) hyperchloremia (>107 mmol/L), 2 (6.5%) hyponatremia (<135 mmol/L), and 5 (16.1%) hypokalemia (<3.5 mmol/L). The median viral load for CHIKV was 1.76 × 10^5^ (IQR 1.17 × 10^4^ to 2.95 × 10^6^) copies/mL. For all patients, test results for dengue viruses (nonstructural protein 1, IgM, and IgG) were negative.

**Table 1:** Patient characteristics and clinical signs and symptoms of disease of 60 pregnant women with chikungunya virus infection, Colombia, 2015

Characteristics	Total, n = 60	Trimester		
		First, n = 5	Second, n = 17	Third, n = 38
Patient age, y, average ± SD	26.4 ± 5.6	27.8 ± 7.5	26.8 ± 5.8	26 ± 5.3
Gestational age of fetus, wks, average ± SD	28.5 ± 8.8	13 ± 5.8	20.6 ± 5.6	34 ± 3.7
Reason for consultation, no. (%)				
Arthralgia	55 (91.7)	5 (100)	17 (100)	33 (86.8)
Fever	21 (35)	0 (0)	5 (29.4)	16 (42.1)
Pruritus	4 (6.7)	0 (0)	0 (0)	4 (10.5)
Headaches	9 (15)	1 (20)	4 (23.5)	4 (10.5)
Rash	9 (15)	4 (80)	5 (29.4)	0 (0)
Signs and symptoms during hospitalization, no. (%)				
Fever	11 (18.3)	0 (0)	0 (0)	11 (28.9)
Polyarthralgia	56 (93.3)	5 (100)	16 (94.1)	35 (92.1)
Headaches	45 (75)	4 (80)	12 (70.6)	29 (76.3)
Rash	52 (86.7)	5 (100)	13 (76.5)	34 (89.5)
Myalgia	46 (76.7)	4 (80)	13 (76.5)	29 (76.3)
Low back pain	28 (46.7)	3 (60)	6 (35.3)	19 (50)
Emesis	3 (5)	0 (0)	1 (5.9)	2 (5.3)
Nausea	11 (18.3)	2 (40)	2 (11.8)	7 (18.4)
Epistaxis	2 (3.3)	0 (0)	0 (0)	2 (5.3)
Gingivorragia	3 (5)	0 (0)	0 (0)	3 (7.9)

We stratified the observed obstetric complications by the trimester in which the acute CHIKV infection occurred. Among women in their first trimester, 1 had a spontaneous abortion; among women in their second trimester, 3 had preeclampsia and 1 was admitted to the ICU because of CHIKV sepsis; and among women in their third trimester, 8 had CHIKV sepsis. The average hospital stay was longer for the women with CHIKV sepsis in their third trimester (4.3 ± 2.7 days) than for pregnant women without CHIKV (2.4 ± 1.2 days). The obstetric pathologies observed during the third trimester were premature rupture of membranes (3/38, 7.9%), intrauterine growth restriction (2/38, 5.3%), preeclampsia (6/38, 15.8%), preterm delivery (1/38, 2.6%), and postdelivery hemorrhage (3/38, 7.9%).

### Intrapartum Period

Fifteen (39.5%) of the 38 patients who sought treatment for chikungunya fever in the third trimester gave birth during the hospitalization: 10 by vaginal delivery and 5 by cesarean section (because of unsatisfactory fetal orientation in the womb). The mean gestational age of their newborns at birth was 38.5 ± 1.08 weeks. For these 15 women, the average number of days from onset of symptoms to labor was 6.3 ± 1.9 days. Vital signs were monitored every 2 hours and platelets and hemoglobin every 24 hours, and no severe thrombocytopenia or anemia occurred. These patients required resuscitation with intravenous crystalloid fluids, with a cumulative fluid balance of 328 ± 657 mL during the obstetric event. Three (20%) women with regular uterine activity had fevers, so the doctors performed tocolysis with nifedipine for 2.3 ± 0.94 days to delay labor and prevent birth during the period of intrapartum fever.

### Intensive Care Unit

Nine (15%) patients required ICU admission, 8 (89%) of which were in their last trimester of pregnancy. Although women in the third trimester comprised 63.3% of the study population, they comprised almost all of the ICU admissions. Seven (78%) of the 9 ICU patients did not have other comorbidities, and 2 (22%) had reported previous cardiac arrhythmias. All 9 women had sepsis, 7 (78%) had the criteria for severe sepsis, and none had septic shock. Bacteria cultures were performed with these patients’ blood samples, and all were negative, ruling out nosocomial bacterial infection. Complications developed in some patients, with postdelivery hemorrhage occurring most frequently (11.1%). At admission to the ICU, lactic acid and base deficit were measured. Of the 9 patients sent to the ICU, mean lactic acid level was 2.6 ± 2.39 mmol/L and base deficit was −5 ± 1.4 mmol/L; 6 (66.7%) patients had hyperlactatemia.

Upon admission to the ICU, all 9 patients received resuscitation with crystalloids (30 mL/kg in 500 mL boluses every 15–30 min), resulting in a fluid balance of 960.3 mL at the end of resuscitation. Three women had hypertension, and 6 had clinical signs of tissue hypoperfusion. The mean APACHE II (Acute Physiology and Chronic Health Evaluation II) score was 10.44 ± 4.71 points, and death was the prognosis for 15% of these patients. All patients had SAMM criteria (Table 2); the main dysfunctions found were renal (33%), vascular (22%), and hepatic (22%). Although the patients had SAMM criteria, no maternal deaths were reported.

**Table 2:** Criteria for severe acute maternal morbidity among 9 pregnant women with chikungunya virus infection who were admitted to intensive care, Colombia, 2015*

Criteria	No. (%) patients
Organ dysfunction†	7 (77.8)
Hepatic	2 (22.2)
Renal	3 (33.3)
Vascular	2 (22.2)
Clinical diagnosis	9 (100)
Severe preeclampsia	3 (33.3)
Severe postpartum hemorrhage	1 (11.1)
Sepsis	9 (100)
Interventions in critical care	9 (100)
Admission to intensive care unit	9 (100)
Transfusions of >3 units of red blood cells	2 (22.2)

*The World Health Organization’s definition for severe acute maternal morbidity was used (*24**,**25*).  †Hepatic dysfunction was defined as hyperbilirubinemia (bilirubin >100 µmol/L or 6 mg/dL). Renal dysfunction was defined as oliguria <400 mL that did not resolve after administration of fluids or diuretics. Vascular dysfunction was defined as hypovolemia requiring transfusion or use of vasoactives.

### Newborn Infants

Of the 15 infants born during their mothers’ acute CHIKV infection, 12 were hospitalized as recommended by the pediatric neonatologist to observe their clinical progress and to rule out vertical transmission. The average duration of hospitalization of newborns was 4 days. Because this event was the department of obstetrics and neonatology’s first experience managing an outbreak of CHIKV in pregnant women, the hospital did not have a protocol to care for newborns born from mothers with CHIKV infections. A decision was made to perform RT-PCR only with newborns of mothers having viremia near the time of delivery (50% of the 12 hospitalized newborns), and all were negative for the CHIKV genome. At physical examination, 5 neonates had no abnormalities, and 1 neonate had a short neck with no internal anatomic abnormalities or problems with mobility. For this neonate, a karyotype was performed, leading to the diagnosis of Turner syndrome. Regarding the laboratory results of the other neonates, 2 (16.7%) of 12 had leukocytosis and 1 (8.3%) of 12 had lymphocytosis. No abnormalities were found with renal, liver, or platelet function tests. No neurologic or cardiovascular abnormalities were observed.

### Postdelivery Follow-up

One year after delivery, we called study participants to perform follow-up of the chronic phase of disease. Only 36 patients (60%) could be contacted; the other 24 patients did not or were not willing to answer our calls. Twenty-three women had no residual symptoms. However, 13 patients (36% of the women who responded to the survey) experienced arthralgia in >2 joints. Of these women, 8 had joint swelling, 7 had erythema, and 4 had myalgia. Nine had an inflammatory pattern that included morning stiffness. The presence of these symptoms indicates these women were experiencing a relapse of acute disease. Joint pain reoccurred 72.6 ± 74.15 days after acute CHIKV infection and persisted for 186.9 ± 85.78 days. Five women required follow-up with a rheumatology specialist.

## Discussion

Our cohort included 60 pregnant women with a diagnosis of CHIKV confirmed by RT-PCR. To enable observation of the behavior of the disease and the obstetric outcomes, all patients with confirmed CHIKV infection were hospitalized, regardless of clinical stability and severity of the infection. In 2015, the Fundación Clínica Valle del Lili did not have an established protocol for the management of pregnant women with CHIKV infection. The most common signs of illness in the patients admitted to the UOHC were polyarthralgia, exanthema, myalgia, headache, and fever. Thiberville et al. conducted a prospective study during the epidemic on Reunión Island that included 54 adults with CHIKV infection (*28*). This study indicated the same signs described in our series. Our analysis also confirms that the clinical manifestations do not vary between nonpregnant adults and pregnant women (*29*).

Previous studies of the nonpregnant population indicated that CHIKV can cause illnesses and deaths associated with sepsis (*30*–*32*). Our study reports 9 pregnant women with CHIKV-induced sepsis, and all required ICU treatment. Seven (77.8%) of those women had severe sepsis. Target-guided resuscitation was performed with crystalloids and vasopressors under strict surveillance of water overload. Among patients with SAMM, the APACHE II score was 10.4 ± 4.7 points, and 15% had a prognosis of death; however, no deaths occurred. These findings suggest that CHIKV infection during pregnancy can cause severe sepsis with organ dysfunction and tissue hypoperfusion.

CHIKV infection appears to have clinical presentations that differ by trimester of pregnancy. Another report related maternal infection with vertical transmission and pregnancy loss (*33*), although these occurrences were uncommon. In our cohort, spontaneous abortion occurred once; however, two thirds of our patients (38/60) were in their third trimester. This high concentration of women in late pregnancy might be explained by the common practice of low-complexity obstetrics centers of Colombia referring high-complexity obstetrics candidates (pregnant women at term or during labor with acute febrile syndrome) to Fundación Valle del Lili’s UOHC.

Chikungunya is a potential risk for neonates born to symptomatic women (*11*,*14*). The most common clinical signs in newborns were fever, irritability, rash, hyperalgesia syndrome, diffuse limb edema, meningoencephalitis, and bullous dermatitis (*8*,*11*). With the Réunion Island outbreak of 2005, Gérardin et al. found that the risk for vertical transmission increased to 50% when maternal viremia was present during delivery (*11*). Furthermore, Ramful et al. found that the risk for mother-to-child transmission increased when the acute infection was documented during the intrapartum period (*12*). These findings suggest that the intrapartum period is the most critical time for vertical transmission. In 2016, a multicenter study occurring in 3 Latin America countries (El Salvador, Colombia, and Dominican Republic) showed vertical transmission rates ranging from 27.7% to 48.3% (*20*).

In our cohort, we could not rule out the transmission of CHIKV infection in all newborns but report that none had clinical signs of congenital infection. We could test only 50% of the newborns with RT-PCR, and all were negative. We delayed labor to prevent birth during the early febrile phase. The average interval from the onset of maternal symptoms to delivery of 6.3 ± 1.4 days might have been enough time for the passive transfer of antibodies to occur to prevent symptomatic infection in the newborn. We strictly monitored these patients for the presence of fever, thrombocytopenia, or intrapartum hemodynamic decompensation; 20% of the patients with intrapartum fever required tocolysis to delay labor. We have used this approach to prevent dengue maternal complications and to reduce dengue vertical transmission at Fundación Valle del Lili. Our findings suggest delaying the birth as long as possible in patients with acute febrile CHIKV infections is necessary, as long as there are no obstetric contraindications. In favor of this perspective, in the Leglet et al. study, 118 of 151 pregnant women with CHIKV infection had a clearance of their viremia before completing gestation, and no cases of vertical transmission were reported (*34*).

We found that the median viral load for CHIKV was 1.76 × 10^5^ (IQR 1.17 × 10^4^ to 2.95 × 10^6^) copies/mL. In 2006, Panning et al. conducted a study with patients who returned to Europe from the Indian Ocean with a diagnosis of CHIKV and reported a mean viral load of 9.85 × 10^7^ copies/mL (*35*).

Although this type of study cannot establish causality, we found a high incidence of preeclampsia (15.7%) in patients with CHIKV. In Colombia, preeclampsia is the leading cause of maternal illness and death; in 2014, a total of 10,499 cases of maternal illnesses were reported, with hypertensive disorders being the main associated cause (60.5% of cases) (*36*). Likewise, hypertensive disorders are one of the main reasons for consultations with the Fundación Valle del Lili (24% of all consultations per year).

The chronic phase of CHIKV has been poorly described in the obstetric population. In our study, 36 women (60%) were followed up 1 year after seeking treatment for acute CHIKV infection, and 13 patients (36%) were found to have episodes of arthralgia in >2 joints (relapsed infections). These findings are consistent with the study by Rodriguez et al., which characterized the chronic phase of CHIKV in an adult population in Colombia and reported that 50% of patients had chronic rheumatologic disorders with an inflammatory pattern (*16*). This observation is consistent with that from our report, in which 5 patients (13.9%) required care by a rheumatology service. Similarly, Rodríguez et al. reported that a higher proportion of chronic pain might occur with some groups, such as women and the elderly (*16*). These findings emphasize the need to further characterize the degree of disability caused by rheumatologic symptoms in this population.

Our findings have methodologic limitations. First, this study involved a small retrospective cohort without a control population, so assessing causality and risk factors was not possible. Second, the hospital used was a reference center for the most serious cases in the region, generating a selection bias in recruited patients and explaining the high incidence of preeclampsia in this series. Third, electronic medical records of hospitalized women were used, which led to a restricted nongeneralizable sample. Fourth, because follow-up of the chronic phase of the disease was 1 year after onset of symptoms, memory bias could have affected our results. Finally, vertical transmission could have been underestimated given that not all neonates were hospitalized and, of those hospitalized, only half had RT-PCR to evaluate for CHIKV infection.

In conclusion, chikungunya is an emerging disease in the Western Hemisphere. All personnel in charge of obstetric populations should be aware of the behavior of CHIKV infection during pregnancy. This study suggests that CHIKV might cause cases of sepsis with hypoperfusion and organ dysfunction. Although the number of neonates potentially exposed was low, good perinatal outcomes without vertical transmission of infection justifies high-complexity obstetric center care of pregnant women, particularly those with signs and symptoms of CHIKV infection near term or at the time of delivery. Finally, the presence of residual pain in our patients suggests the need for follow-up throughout the first year after infection.

Technical Appendix Survey used to assess symptoms of women 1 year after infection with chikungunya virus during pregnancy, Colombia.
