# Supplementary material for: Pregnant Women Hospitalized with Chikungunya Virus Infection, Colombia, 2015
Source: Emerg Infect Dis. 2017 Nov;23(11):1777–83. doi: 10.3201/eid2311.170480 (PMC5652420; doi:10.3201/eid2311.170480)
Supplement: Technical Appendix — Survey used to assess symptoms of women 1 year after infection with chikungunya virus during pregnancy, Colombia. [file 17-0480-Techapp-s1.pdf]

Article DOI: https://doi.org/10.3201/eid2311.170480

Pregnant Women Hospitalized with Chikungunya Virus Infection, Colombia, 2015

Technical Appendix

Technical Appendix Table. Survey used to assess symptoms of women 1 year after infection with CHIKV during pregnancy, Colombia

Question	Answer
1. After being diagnosed with CHIKV, did you experienced joint pain?	0. No<br>1. Yes
2. How much time elapsed between diagnosis and the onset of joint pain?	Open-ended answer, quantified according to time in days
3. How long have you been experiencing joint pain?	Open-ended answer, quantified according to time in days. The answer “I do not know” was included
4. In how many joints have you been experiencing pain?	Open-ended answer. The answer “I do not know” was included.
5. Name which joints have experienced pain.	Open-ended answer. The answer “I do not know” was included.
6. When you have joint pain, do they turn red?	0. No<br>1. Yes
7. When you have joint pain, when you are waking up in the morning, do you experience joint stiffness?	0. No<br>1. Yes
8. When you have joint pain, do you experience joint swelling?	0. No<br>1. Yes
9. When you have joint pain, do you experience muscle pain?	0. No<br>1. Yes
10. Have you consulted to healthcare services regarding joint pain?	0. No<br>1. Yes
11. If question 11 has a positive answer. How much time elapsed between diagnosis and doctor consultation?	Open-ended answer. The answer “I do not know” was included.
12. Have you consulted to a rheumatologist regarding the joint pain?	0. No<br>1. Yes
13. Please name which drugs you have used to control your joint pain.	Open-ended answer. The answer “I do not know” was included.

*CHIKV, chikungunya virus.
